# An investigation of multidisciplinary complex health care interventions - steps towards an integrative treatment model in the rehabilitation of People with Multiple Sclerosis

**DOI:** 10.1186/1472-6882-12-50

**Published:** 2012-04-23

**Authors:** Lasse Skovgaard, Liv Bjerre, Niels Haahr, Charlotte Paterson, Finn Boesen, Michael Nissen, Mai-Britt Ottesen, Christina Mortensen, Anette Olsen, Søren Borch, Birthe K Mortensen, Gudrun Aa Rasmussen, Kirsten Sietam, Frank Staalkjær, Karin Pedersen, Kirsten Søndermark

**Affiliations:** 1 The Danish MS Society, Valby, Denmark; 2 Department of Public Health Research, University of Copenhagen, Copenhagen, Denmark; 3 The Danish MS Hospital, Haslev, Denmark; 4 Institute of Health Services Research, Peninsula Medical School, University of Exeter, Exeter, UK; 5 The National Research Centre on Complementary and Alternative Medicine (NAFKAM), University of Tromsø, Tromsø, Norway

## Abstract

**Background:**

The Danish Multiple Sclerosis Society initiated a large-scale bridge building and integrative treatment project to take place from 2004–2010 at a specialized Multiple Sclerosis (MS) hospital. In this project, a team of five conventional health care practitioners and five alternative practitioners was set up to work together in developing and offering individualized treatments to 200 people with MS. The purpose of this paper is to present results from the six year treatment collaboration process regarding the development of an integrative treatment model.

**Discussion:**

The collaborative work towards an integrative treatment model for people with MS, involved six steps: 1) Working with an initial model 2) Unfolding the different treatment philosophies 3) Discussing the elements of the Intervention-Mechanism-Context-Outcome-scheme (the IMCO-scheme) 4) Phrasing the common assumptions for an integrative MS program theory 5) Developing the integrative MS program theory 6) Building the integrative MS treatment model. The model includes important elements of the different treatment philosophies represented in the team and thereby describes a common understanding of the complexity of the courses of treatment.

**Summary:**

An integrative team of practitioners has developed an integrative model for combined treatments of People with Multiple Sclerosis. The model unites different treatment philosophies and focuses on process-oriented factors and the strengthening of the patients’ resources and competences on a physical, an emotional and a cognitive level.

## Background

Across the world, a wide variety of projects have sought to combine conventional and alternative medical (CAM) treatments. These include initiatives by individual physicians, hospital based collaborations between conventional and alternative health care practitioners, collaborations between hospitals and private clinics, and integrative initiatives within primary healthcare [1-12]. Despite the efforts to integrate CAM with conventional health care interventions and to initiate bridge building between conventional and CAM providers, focus has rarely been put on exploring the complexity of the courses of treatment that results from such combined intervention. In the light of these issues, the Danish Multiple Sclerosis Society initiated a large-scale bridge building and integrative treatment project to take place from 2004–2010 at a specialized MS hospital – the MS Treatment Team Project. In this project, a team of five conventional practitioners (a neurologist, a psychologist, a physical therapist, an occupational therapist and a nurse) and five CAM practitioners (an acupuncturist, a nutritional therapist, a homeopath, a cranio sacral therapist and a reflexologist) was set up to work together in developing and offering individualized treatments to 200 People with Multiple Sclerosis (PwMS) (results regarding the treatment outcomes have been published in [13-15]).

One of the main purposes of the research project was to investigate the six year collaboration process within the team of practitioners (overall results have been published in [16]). During this period, the ten practitioners worked towards a common understanding of the complexity of the courses of treatment which resulted in the description of a unified program theory (intervention theory). The purpose of this paper, written at the end of the project, is to present an overview of the main steps in the collaboration process that resulted in the construction of an integrative MS treatment model, providing an over-arching framework for integrative treatment within the area of MS care.

## Discussion

### Working with program theory

The extent to which a health care intervention causes or facilitates health-related change is a key question in research and practice. One of the major challenges in integrative treatment efforts is the complexity in the courses of disease and treatment. It has been argued that the core question “what works?” should be rephrased in an extended form, “what works, for whom, when, where, and why, and from whose perspectives”? [17].

Program theory (intervention theory, logic modelling) was originally developed to research and evaluate social policies and programs [18-22]. It conceptualises outcome as linked to both mechanism and context. Used as a basis for constructing conceptual models of complex health care interventions, program theory ensures that such models include not only the *intervention* and the *outcomes* but also explicitly represents the components and dynamic of the *process* and the social and cultural *context*s. It pushes us to unpack different actors’ (biomedical practitioners, complementary practitioners, patients, health service managers) theories and assumptions about how an intervention (programme) might work and to understand more about how conflicts between, or better communication about, these different assumptions affect outcome. Program theory is also a tool to help us reflect on blind spots or failures in the connections between purposes, interventions, processes the interventions generate, contexts that facilitate or inhibit treatment effects, as well as expected or obtained short-term and long-term effects [17,20].

### Step 1: The initial model

**Figure 1:**
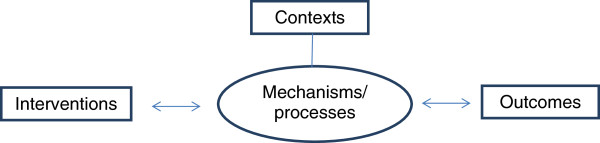
The first IMCO-model.

A simple model was initially developed on the basis of program theory, illustrating four basic elements of an integrated care effort. As shown in Figure 1, both contexts and processes were seen as potentially important aspects of a treatment course and were seen as functioning in synergetic coherence with the interventions and the outcomes.

### Step 2: Unfolding the different treatment philosophies

With the purpose of unfolding basic assumptions within the different treatment philosophies represented in the MS Treatment Team Project, a specific tool called an IMCO-scheme, was developed on the basis of program theory. The IMCO-scheme (Table 1) – described the Interventions, Mechanisms, Contexts and Outcomes of the respective treatments and was used for the purpose of facilitating knowledge sharing and mutual understanding in the team [23]. Another intention of the scheme was to facilitate confrontation and make conflicts visible, explicit and legitimate. In the initial phase of the project, every practitioner was asked to fill out the IMCO-scheme for their respective treatment models by outlining typical Interventions, the presumed Mechanism of action, as well as Contextual factors, which could possibly affect Outcomes positively or negatively.

**Table 1:** Definitions of the four IMCO elements – examples from four practitioners in the team, regarding the same MS patient

	**Intervention**	**Mechanisms**	**Contexts**	**Outcomes- expected**	**Outcomes- obtained this far**
**Medical doctor**	*“Tablet Carduran 8 mg daily”*	“*Adrenergic Alfa-receptor blocking effect on post synaptic Alfa 1-receptors, whereby the urethrale resistance is reduced*”	*“Compliance promotes the treatment”*	*“Improved bladder discharge and reduction of pollakiuria”*	*“Reduction of frequent urination urge”*
**Physical therapist**	*“Therapeutic horseback riding”*	*“Strengthens the balance and stimulates the walking pattern”*	*“The treatment is promoted by the patient’s motivation and inhibited by the risk of skin chafing due to low body weight”*	*“Improvement of balance and walking pattern”*	*“Walking pattern is more relaxed”*
**Nutritional therapist**	*“An MS diet with a high intake of vegetables, fibres and fish and a low intake of saturated fatty acids and sugar”*	*“A lower intake of saturated fatty acids reduces the level of inflammatory eicosaniods and thereby limits the inflammatory pain”*	*“The patient needs to enter a positive circle where a changed diet provides the energy needed to continue a positive process. Smoking inhibites the treatment”*	*“Less tiredness, less pain and more energy”*	*“The patient has more energy, has regained a feeling of freedom and optimism. The mood has been improved”*
**Homeopath**	*“Agaricus C200, Arnica C30, Natrium, Muriaticum C200 and 1 M”*	*“The homeopathic remedies stimulate the patient’s self healing powers at different levels”*	*“The patient’s participation, motivation and willingness to cooperate promotes the treatment. The patient is emotionally burdened, which inhibites the treatment”*	*“Improvements on the emotional level, better accept of own situation. Improvement of walk and balance”*	*“Improvements on the emotional level. More energy mentally and physically. Better physical endurance. Improvement of spasms in legs”*

The schemes, which were filled out by every practitioner, were subsequently distributed to the other practitioners and differences as well as similarities in the ten treatment approaches were discussed in seminars within the team of practitioners and researchers [23-26]. Throughout the project process, four seminars were conducted yearly, where practitioners and researchers met to discuss the collaboration within the team as well as specific patient cases and continuous research results. During the project, the IMCO-scheme was used as a tool to analyse and reflect upon specific patient cases and the practitioners were asked to relate to the expected outcomes from the treatments as well as to the actual outcomes obtained at that specific point in the individual courses of treatment.

In Table 1 examples from four of the ten practitioners’ IMCO schemes are presented, regarding a specific MS patient. The examples originate from a seminar held midway through the six-year collaboration process and show differences as well as accordance between the treatment modalities in the team.

### Step 3: Discussing the elements of the IMCO-scheme

As shown in Table 1, differences and agreements were found within the treatment team regarding the elements of Intervention, Mechanisms, Contexts and Outcomes. Many differences that were detected pertained to terminology. Perceptions of specific health-related mechanisms were one of the key differences between the practitioners. Nevertheless, the study found fundamental similarities and agreements with regard to the overall perspectives and strategies of MS care. Consensus was established in relation to fundamental aspects of the IMCO elements during the collaboration process. Details regarding this process are presented in [16,23,27-29]. These differences and similarities between the ten practitioners’ treatment assumptions regarding the four IMCO elements were discussed in the team for the purpose of achieving a common frame of understanding integrative treatment of people with MS. Hence the focus was to find common features in the individual perceptions and understandings among the ten practitioners and hereby build the foundation of a program theory for an integrative MS treatment.

### Step 4: Phrasing the common assumptions for an integrative MS program theory

On the basis of extensive work with the four elements of the IMCO-scheme, the ten practitioners phrased seven common assumptions and statements regarding the integrative MS treatment. These common assumptions and statements constitute basic elements of a unified treatment philosophy:

• The combined interventions aim at affecting the patient on the physical as well as on the emotional and cognitive area

• The three areas work together in a dynamic interaction

• The purpose is at first to strengthen the patient’s resources and competences

• Strengthening of the patient’s resources and competences aims to initiate health promoting processes

• The patient’s own efforts/participation is an important treatment goal, as is to strengthen the patient’s ability to contribute actively to the courses of treatment

• The patient’s efforts in connection to concrete treatment initiatives as well as the patient’s ability and will to interact in processes of change constitute important aspects of the treatment

• The contexts of the treatment have significant importance for the course of treatment and the achieved treatment results

### Step 5: Developing the integrative MS program theory

Based on these overall common assumptions, the ten practitioners described in an IMCO-scheme the common perceptions and understandings about Interventions, Mechanisms, Contexts and Outcomes, and thereby developed the foundations of an integrated MS program theory (Table 2).

**Table 2:** The integrative MS program theory

**Intervention**	**Mechanisms**	**Contexts**	**Outcomes**
**The combined interventions are characterised by:**	**The combined interventions aim fundamentally at:**	**Contextual factors are seen as important in prohibiting or enhancing the generation of positive treatment results:**	**Positive treatment results for PwMs are defined as:**
- individual diagnostics and treatment efforts	- strengthening the patient’s resources and competences on a physical, an emotional and a cognitive level	- social, cultural, economical and political relations	-clinical effects, independent of the patient’s feedback
- involvement of the patient’s goals (and possible modifications of these during the course of treatment)	And thereby	- other treatments used	-experienced outcomes, dependent of the patient’s feedback
- coordination of the interventions involved	- initiate and maintain health- promoting processes	- practitioner-settings	Outcomes are obtained through a dynamic process and can therefore not be reduced to the attainment of pre-defined treatment goals
		- the patient’s own effort	
		- the patient’s motivation	

### Step 6: Building the integrative MS treatment model

**Figure 2:**
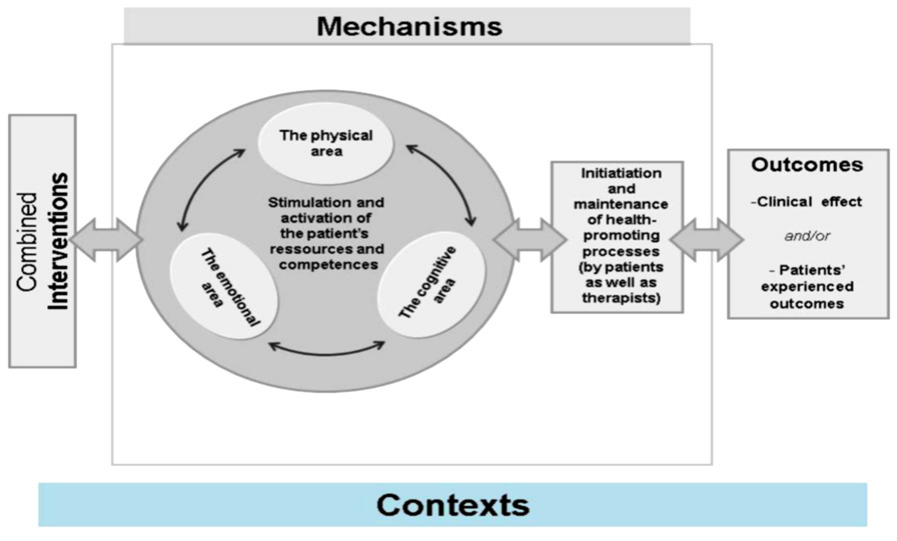
The integrative MS treatment model.

Using the integrative program theory, the team, in collaboration with the researchers, developed a conceptual model (Figure 2), which illustrates the common foundations of the integrative program theory and thereby presents a unified treatment philosophy among the ten practitioners:

Figure 2 illustrates that the integrated treatment philosophy largely focuses on process-oriented factors in the courses of treatment. The strengthening of the patient’s resources and competences is pointed out by the practitioners as a fundamental treatment goal. Regarding treatment results, focus is put on the outcomes that occur over time when strengthened resources and competences work in dynamic interaction with the patient’s own efforts under the right contextual circumstances.

### Applications and implications

Program theory has been of great value in The MS Treatment Team Project. It has proved to be a very useful tool in the process of describing basic treatment assumptions and thereby identifying differences as well as similarities within different treatment philosophies. It has also been valuable in the process of developing the foundations of an integrative and unified treatment philosophy, on which a conceptual model could be built. We anticipate that the work presented in this paper will be useful to other treatment centres who are seeking to provide integrated care and that the integrative program theory as well as the conceptual model will be further developed and elaborated by them.

Having worked with program theory in connection with The MS Treatment Team Project, we believe that the use of program theory within health care and health care research may be useful:

• As a tool to promote and qualify interdisciplinary collaboration

• As a tool to strengthen collaboration between practitioner and patient

• As a tool to investigate and understand patients’ basic treatment assumptions and thereby provide a better understanding of their behaviour

• As a tool to investigate and develop common terminologies

• As a tool to specify limitations in interdisciplinary collaboration

• As a tool to obtain better understanding of complexities in treatment interventions and courses of treatment for people with chronic illness

## Summary

In this paper we have outlined how program theory has been used to investigate complexities in MS treatment. We have shown how it has been used to discuss, compare and unite the different treatment perspectives within an integrative team of practitioners. We have given examples of its use in defining a common understanding of the concepts of Intervention, Mechanism, Context and Outcome. The work has resulted in the development and description of basic elements of a unified and integrated model for integrative treatments of People with Multiple Sclerosis that focuses on process-oriented factors and the strengthening of the patients’ resources and competences on a physical, an emotional and a cognitive level.

## Competing interests

The authors declare that they have no competing interests.

## Authors’ contributions

LS, LB, NH, CP and LL helped conceive, design and draft this manuscript. FB, MN, MO, CM, AO, SB, BKM, GAR, KS, FS, KP and KS helped conceive, design and provided critical edits to this manuscript. All authors made contributions to the acquisition, analysis and interpretation of data. All authors read and approved the final manuscript.

## Pre-publication history

The pre-publication history for this paper can be accessed here:

http://www.biomedcentral.com/1472-6882/12/50/prepub
